# A Web of Things-Based Emerging Sensor Network Architecture for Smart Control Systems

**DOI:** 10.3390/s17020332

**Published:** 2017-02-09

**Authors:** Murad Khan, Bhagya Nathali Silva, Kijun Han

**Affiliations:** School of Computer Science and Engineering, Kyungpook National University, Daegu 41566, Korea; mkhan@netopia.knu.ac.kr (M.K.); nathalis@netopia.knu.ac.kr (B.N.S.)

**Keywords:** Web of Things, smart home, smart control systems, emerging sensor networks

## Abstract

The Web of Things (WoT) plays an important role in the representation of the objects connected to the Internet of Things in a more transparent and effective way. Thus, it enables seamless and ubiquitous web communication between users and the smart things. Considering the importance of WoT, we propose a WoT-based emerging sensor network (WoT-ESN), which collects data from sensors, routes sensor data to the web, and integrate smart things into the web employing a representational state transfer (REST) architecture. A smart home scenario is introduced to evaluate the proposed WoT-ESN architecture. The smart home scenario is tested through computer simulation of the energy consumption of various household appliances, device discovery, and response time performance. The simulation results show that the proposed scheme significantly optimizes the energy consumption of the household appliances and the response time of the appliances.

## 1. Introduction

Pervasive and ubiquitous computing has favored the integration of physical objects into the digital world, which has been in the spotlight of the research community for the past few decades. The advancements in embedded device technology have resulted in the emergence of the smart things concept, which has boosted the notion of connecting everyday objects to the existing networks. The tremendous growth of smart things has led to a major breakthrough creating the Internet of Things (IoT) as the third wave of the Web after the Static Pages Web and the Social Networking Web. The IoT is expanding steadily thanks to the use of novel technologies, i.e., Radio Frequency Identification (RFID) and Wireless Sensor Networks (WSNs) [1]. RFID tags enhance the operational performance in supply chain management and warehouses. Similarly, WSN improves the smart home and smart city applications with the aid of the connected sensors. In addition, the sensor networks successfully implement environment monitoring applications. Nowadays, both academic and industrial experts are focused on improving and inventing low power communication protocols to enable effective communication among these devices. Bluetooth, IEEE 802.15.4, IPv6 over Low-Power Wireless Personal Area Network (6LoWPAN), and Constrained Application Protocol (CoAP) are some of the assisting communication protocols [2]. Although these protocols can facilitate the integration of smart things at the network layer, at the application layer that is still a challenging task due to platform incompatibilities. 

Recent studies have identified the need for a common platform to integrate heterogeneous smart things. However, building a universal platform for smart things is a tedious task. Sensors networks are emerging every day and are considered the backbone of the IoT. Moreover, the transfer of information over the existing WSNs based on the current communication and routing protocols has several limitations, i.e., integration with the new paradigms such as smart homes and smart cities, dynamic routing issues and power management of the sensors. Therefore, refining a widely accepted platform instead of developing a new platform seems to be an advantageous approach. The Internet is a widespread network of computers that facilitates interoperability across heterogeneous hardware and software. In general, the Web consolidates open and simple standards to build efficient and scalable systems on top of the Internet [3]. However, streaming the data from the top of the Internet to the bottom is still a challenging job [4,5]. Thus, an efficient and concise modification of the existing WSN protocols is necessary. The ubiquity and cross-platform interoperability make the Web an excellent fit for the universal platform demands. The Web is used to create scalable and collaborating applications. Continuous integration of smart things into the Internet has further confirmed the suitability of the Web as the mutual integration platform. Thereupon, the WoT arises as a novel paradigm that adapts conventional Web-associated technologies into the IoT. Indeed, WoT-ESN is an adaptation of the IoT which integrates smart things (all those objects connected to sensors) into the network layer as well as the application layer. The concept of WoT is entrusted to connect everything to the Web [6]. Web services reveal the data, in order to reuse information across the web. The integration of smart things into the Web is beneficial in many ways, i.e., faster sensing, increased sensing accuracy, transfer of the data in efficient ways avoiding faulty nodes, consideration of the positioning of the sensor nodes and cost efficiency [7,8]. In addition, unlike the WoT, WoT-ESN uses reliable and stable Web architecture paradigms to integrate smart things (sensors, actuators, coordinators, etc.) into the Web [9]. For example, WoT-ESN utilizes the well-established Representational State Transfer as the architecture style to design networked applications [10]. It collaborates with Hypertext Transfer Protocol (HTTP) as the application layer protocol. Consequent to the reuse of HTTP, smart things become capable of acting like any other resource on the Web. The smart objects exchange HTTP requests and responses in order to communicate with other components connected to the Web. In fact, WoT operates on the application level of the Open System Interconnection model dealing with applications, services, and data, while offering a high level of abstraction [11]. Thus, WoT-ESN is able to connect various data and services despite differences in the actual transport protocols. The abstraction of lower level protocols is advantageous in various aspects. The Web acts as the common platform for applications throughout the Internet. Similarly, WoT-ESN acts as the universal sensor platform, which facilitates interaction among heterogeneous sensor devices. Moreover, the abstraction hides the numerous transport protocols that are utilized at lower levels. Accordingly, the applications at hand can receive more attention from the developers instead of panicking over the underlying protocols of the devices to be integrated. The WoT architecture assures the complete integration into the Web as of all the devices follow the same Web standards. Thereupon, the embedded devices can use a Web API to communicate with each other. In the proposed WoT-ESN scheme, we have specifically selected RESTful Web APIs. REST identifies services on the Web using Uniform Resource Identifier (URI) using HTTP as the application protocol. Upon getting a URI, each device and service can be exchanged, referenced on websites, and can be bookmarked [3]. In other words, smart things are explored simply via Web browsing. A browser can control the functionalities of a smart thing by acting as a mediator between the actual physical object and the user. Moreover, the developers can apply Web languages, tools, and technologies to develop the applications needed to control the sensors attached to various things. In addition, the proposed WoT-ESN architecture benefits smart things by facilitating caching, indexing, searching, locating, and load balancing. 

The Web has transformed the nature of computing throughout for the past few decades. Similarly, IoT technologies are changing with the notion of WoT. The emergence of WoT has divided the research focus of experts into many fields. The Web is expected to be filled with real-time data and services from the real world along with the expansion of WoT. The real-world devices and sensors generate a massive amount of real-time information compared to the Static Page Web. Since the WoT consists of real world devices, it is highly dynamic and frequently changes in status in accordance with the connected devices. Thus, the WoT search engines should discover real-time data and services efficiently. In [12], a discovery service was proposed using RESTful interfaces [13]. In addition, the optimization of IPv6 based 6LoWPAN protocol has become popular among the research community. The concern about studying smart things has pioneered the study of WoT applications. Consequently, current research is focused on WoT applications for eHealth, smart homes, and sustainable environments [14]. Home automation is widely used for central controlling of lighting, heating, ventilation and air conditioning appliances (HVAC) and security locks. Due to the heterogeneity of devices, providing a common platform for the smart home devices has been a challenging task. However, the concept of integrating smart things into the Web is driving towards a promising future, which can overcome the difficulties of heterogeneity. The smart home devices can be exposed to the users via the Web with the aid of the WoT, thereby enhancing the availability of smart home services, while complying with the needed dynamicity. Therefore, a smart home architecture integrated with WoT mitigates the effects of heterogeneity and provides access to real-time data and services over the Web.

In the proposed WoT-ESN scheme, smart home services are integrated with the Web using a smart gateway. The scheme consists of two components: (1) smart home services at the smart home end and (2) RESTful smart home services at the WoT end. The smart home services aim to enhance the energy consumption efficiency and to reduce the adverse effects of coexistence interference. The smart home’s operations are semi-automated to optimize the energy consumption. This provides a real-time decision-making mechanism to generate necessary events to save energy. However, the user has the sole authority over approving or rejecting the generated events. Meanwhile, a human machine interface (HMI) is embedded into the decision-making system to approve/reject generated events at the absence of the user. The events are notified to the user and the system waits for a time $\alpha_{T}$ for a user response. If the user response is not received within the predefined time, the HMI approves/rejects the generated event in accordance with the previous records. The smart home services are exposed to the Web via RESTful APIs. Remote users can control the smart home operations via the proposed smart gateway. It acts as the bridge between smart things and the Internet. Furthermore, it performs the service translation of the proprietary protocols of smart home devices and sensors, since the services cannot be exposed over the Web using proprietary protocols such as ZigBee The RESTful architecture binds URI to each functionality of smart things. Thus, the remote smart home user can request smart home services via the Web. 

The rest of the paper is organized as follows: Section 2 presents a brief overview of the similar architectures and methods used in the field of WoT. Section 3 illustrates the proposed scheme in detail. The simulation and results are discussed in Section 4. Finally, the conclusion is given in Section 5.

## 2. Related Work

The WoT provides cost effective and fast services with high-speed connectivity integrating heterogeneous wireless networks technologies. Similarly, the introduction of 5G technology will boost the communication among smart objects as well as will satisfy the high data rate requirements. Moreover, the always-connected services problem is significantly addressed by the macro and femtocell technologies that are widely used in enabling network connectivity for indoor communications. However, the integration of WoT in such heterogeneous technologies is still a challenging job. The smart objects are mainly controlled through a sensor which is operated on batteries. Thus, enabling energy-aware communications in such environments must be based on energy-efficient protocols [15,16]. The z-wave short range wireless technology was introduced in [17] to address the home automation in an energy efficient and cost effective way. However, the z-wave technology does not specify the interoperability with the Internet protocols. Therefore, a dedicated gateway is required to translate the z-wave protocol format into an acceptable format for the Internet protocols [17]. A similar work has been done by the KNX protocols by translating the messages down from transport layer up to the application layer using a dedicated gateway. However, z-wave technology is mainly based on ZigBee technology which suffers highly from crowded frequency and compatibility issues, thus, making it less effective for constrained smart objects. Keeping these issues in mind, researchers have proposed several techniques and paradigms to establish seamless communication among smart objects. Thus, the motto of the WoT is to design a generic and well-established architecture to provide ubiquity connectivity between the objects and the Web [7,18]. A major breakthrough was made by Fielding et al. by in integrating smart objects with the Web using REST architecture with the HTTP protocol [19]. However, they did not consider the topology and localization of sensor nodes in integrating the smart things with the Web [20,21]. Similarly, several other protocols have been used for specific purposes such as Extensible Pessaging and Presence Protocols (XMPP) for short and instant messaging and so on. However, HTTP is the de facto protocol mainly used for RESTful applications. Moreover, it is also considered as a sub-optimal protocol for smart objects and specifically those objects which operate on batteries. The HTTP protocol always relies on a long header format to establish TCP communication and also a request/response structure which requires high memory and processing time. Therefore, these issues make HTTP a poor match for the constrained oriented applications [13]. In order to address the aforementioned issues in HTTP protocols, the IETF develop a constrained RESTful group (CORE). The primary task of CORE is to design a constrained oriented structure for CoAP. The main functionality of COAP is to transfer the data from smart objects in a WoT environment with the least possible power and processing [22]. CoAP also supports a limited set of REST requests such as GET, POST and DELETE. Moreover, the caching of resource and device discovery is also carried out using the above requests method. The CoAP mainly uses UDP protocol for transmission. However, the reliability is achieved through a built-in retransmission mechanism. In general, CoAP has many advantages over HTTP in the case of using it for constrained oriented applications [23,24]. The major advantages among those are given below:

1. A UDP protocol along with a header length of 10–20 bytes reduces the delay and battery consumption.
2. The asynchronous push services enable the smart objects with the functionality of sending information of its state change. The smart objects remain in asleep state until and unless it has to send data. Thus, reducing the power consumption.
3. A minimum number of REST requests reduces the complexity of the system. Moreover, it reduces the hardware requirements compare to the HTTP protocol.

CoAP efficiently enhances the transmission capabilities by simplifying the existing architecture of the HTTP protocol. Depending on the implementation, CoAP requires 8–10 lesser bytes compared to HTTP [25]. Moreover, it is very easy to translate and map the HTTP services and functionalities to CoAP [26]. Keeping the above advantages in mind, CoAP is being progressively used by various firms for enabling constrained oriented services in the WoT. However, the standardization of CoAP is still not finalized. Various firms such as INRIA, Lulea, Ericsson, etc. are testing CoAP in the context of implementation and interoperability issues. Moreover, very few products such as Sensinode’s Nano service use CoAP in real time implementations. However which protocol will be used as a standard for WoT communication is not yet finalized. Moreover, if CoAP does not succeed in the future for complex applications and heterogeneous communications, then it is difficult to present it as a standard. Similarly, CoAP may fail like the WAP protocol that was designed for a similar purpose [27]. However, we cannot come up with a final answer in this regard yet because CoAP is still an emerging protocol for WoT applications. The integration of CoAP with UDP is still a challenging job. Several research studies are presented to efficiently integrate CoAP with the UDP sockets. For instance, Kovatsch et al. presented a model based on linking low-end devices with a cloud-based application server [28]. The proposed model was based on the RESTful programming and CoAP protocol. The entire proposed model is based on Java scripts controlling the flow of data from resources to the cloud servers. Similarly, a Peer to Peer (P2P) and parallel implementation of resources with the Web have been developed in [29,30], respectively. Moreover, a Java Network Launching protocol is developed to support centralization of the application instead of distribution and installation. The Java web start applications are very simple and transparent to the end user. Moreover, an end user can perform downloading and installation in just one step. However, such applications directly depend on the respective browsers. Therefore, it is essential to provide the libraries of the browser which can be used to run such scripts and plugins. In addition, Javascript applications require high-speed data transfer and servers because running multiple scripts at once imposes a delay in the process of requesting the information from a sensor mode or smart object.

## 3. WoT-ESN over REST Architecture

In general, Web services have emerged widely during the last two decades, and the integration of Web services with the physical things have shown prominent improvements and developments in the recent past. Several architectures were proposed to enable Web services to interact with the existing physical objects. They have mainly categorized into two groups, i.e., (1) Big Web services and (2) RESTful Web services. In the case of Big Web services or WS_*, interested readers are referred to [31]. The RESTful Web services are becoming popular for the Web connectivity of heterogeneous smart devices [32]. REST is an architecture which is mainly used to explain the workings of HTTP as an application layer protocol. However, the working mechanism of the HTTP is explained in a broader sense separating the traditional use of HTTP from the Web services of physical things. Similarly, RESTful is said to be an Application Programming Interface (API) supporting the REST style. Apart from explaining the basic concept of the REST architecture, many researchers are working on designing Resource Oriented Architectures (ROAs) based on REST. One of the main reasons for employing REST for ROA is its simple architecture and great compatibility with physical and embedded devices. A ROA based on REST follows four basic concepts that are given below:

- Resources: Anything able to provide a service and have the ability to attach with another resource.
- Uniform Resource Identifiers: Naming a resource helps in addressing and discovering a resource over the Web. The URI is used to uniquely identify a resource.
- Linking Resources: The Web provides the structure for linking via hyperlink functionality. This functionality helps to connect resources with each other over the Web. The RESTful API uses this functionality for connectivity.
- Representation of Resources: The restful API uses the XHTML format for representing resources over the Web because of its smooth readability and support for browsing.

REST also uses various kinds of HTTP methods to manipulate resources. However, the following four are very commonly used methods:

1. GET: The GET method retrieves the current state of a resource. For example, sending a GET request with a resource URI (http://.../MS/coordinator1/sensor2_1/fan1/energy) will return the energy consumption of the fan in a smart home.
2. POST: The POST method is used to change the state of a household appliance. For example, a switch off request (http://.../MS/coordinator2/sensor2_2/fan2/Turnoff) is used to switch off a household appliance
3. PUT: The PUT method is used to insert a new resource or modify the existing one. Whenever a new household appliance is added to the smart home, a PUT method is called upon to add it to the resource list.
4. DELETE: Whenever a household appliance is removed from the house. It is important to delete it from the resources list using DELETE method.

The RESTful API is a more convenient and simple solution for the next generations of constraint oriented applications. RESTful APIs are also supported by the HTTP protocol and thus a researcher has a choice of a wide HTTP libraries and clients.

## 4. Proposed Framework

This paper proposes a realistic WoT architecture incorporating a smart gateway to expose smart services over the Web. In this paper, we present a use case scenario based on a smart home to present the service representation over the Web. The proposed architecture works in two parts, i.e.: (1) smart home services (as a use case scenario) and (2) service representation over the Web. An overview of the proposed framework is given in the following section.

### 4.1. Overview of the Proposed Framework

**Figure 1:**
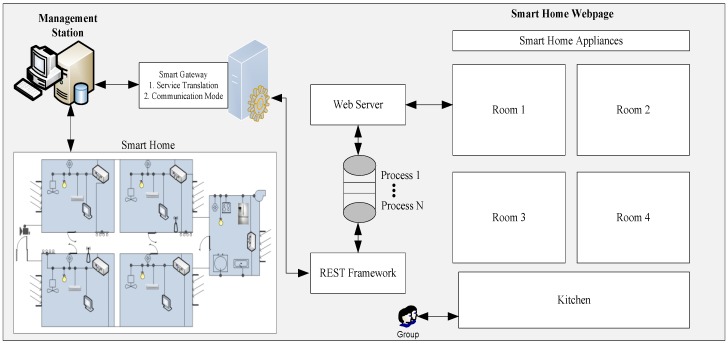
Proposed smart home architecture based on WoT-ESN.

The household appliances are fixed at specific places and they are discovered with the help of specific ids assigned to the sensors that are controlling the appliance. Each new appliance is first registered with a sensor in the MS. Therefore, every HTTP request for an appliance always passes through the MS. Appliance registration in the MS reduces the performance degradation caused by the device discovery process. In order to make the proposed framework work as fast as possible, the proposed RESTful API always communicates with the MS. The MS works as a bridge between the web and the household appliances. Apart from the household appliances control, the working of the RESTful API framework is divided into two modules, i.e.: (1) Process Management Module and (2) Presentation Module. Multiple requests for a single household appliance are divided into multiple processes. Similarly, each process is assigned an ID that distinguishes the respective household appliance/s or sensors deployed in the WoT-ESN. A process is alive until the household device is functioning properly. Once the household appliance is removed from the home, the process terminates autonomously. Whenever a client generates a request, the process of the respective household appliance redirects the request to the resource repository available in the MS. This gives the user many options to retrieve information. For example, if a client requests the energy consumption information of a household appliance, the process shows monthly energy consumption and current energy consumption. Thus, the home users can customize the information to be displayed from the process management. Moreover, multiple clients can generate more than one process for a single household appliance. However, a smart home user can only access services of his own home WoT-ESN. The multi-processing functionality supports multi-user environments, while facilitating a smooth and user-friendly environment for the WoT-ESN. Moreover, the presentation module provides accessibility to the RESTful API from the Web. The smart home users can use any web-browser to view the status of their smart home WoT-ESN owing to the integration of RESTful WoT. Each household appliance is represented as a hyperlink with the name of the appliance. The user can explore the desire services by clicking the links of the respective appliance. The entire web architecture of the smart home is shown in Figure 1.

### 4.2. Smart Home Services

Energy saving has become the utmost goal of current smart home architectures due to the dramatic increase in energy consumption, which has led to a relative increase in the monetary value of energy. Thus, the proposed smart home environment aims to improve the energy consumption efficiency. In this context, the residents’ routines influence the energy consumption demands of the household. The smart home consists of a Device Control Unit (DCU) and an MS. The DCU is implemented to operate in a semi-automated manner. The user switches on the appliances manually and the DCU is capable of switching them off in response to commands from the MS. The DCU contains non-IP appliances and sensors that create the smart home ESN. It includes a collection of ZigBee sensors, which are deployed in the smart home in order to gather environmental parameters and energy consumption parameters. Each appliance in the smart home is attached to a ZigBee sensor to ensure efficient energy management. Each ZigBee sensor is assigned an identification number to distinguish it uniquely. Energy efficiency of the sensor nodes is another key concern of the proposed architecture. Thus, ZigBee sensors are connected to ZigBee coordinators, which are placed in each room and in the kitchen. Mindful coordinator placement reduces the packet loss due to interference caused by coexistence and distance between the sensors and the MS. In addition to the interference management, coordinator placement helps ensure the energy efficiency of the sensor nodes of WoT-ESN. In fact, data transmission from distant nodes consumes more energy compared to proximal nodes. The coordinator placement reduces unnecessary hops during transmission, while improving the energy efficiency of WoT-ESN. The functioning time of a particular appliance determines its energy consumption. Therefore, the MS records the switch on time of devices, once residents turn them ON manually. Since, the appliances are controlled in accordance with user behaviors the DCU periodically checks the user presence at a particular location in the smart home. Whenever the user is not present, all active appliances in that location are turned off to avoid energy wastage. Consequently, the MS records the switched off time of the corresponding appliances. 

The MS handles the communication between users and DCU via an event management system. The MS empowers the smart home performance by processing collected data, organizing data, generating events and performing the corresponding actions. The MS collects and processes the energy consumption of each appliance and stores the records for a period of 30 days. These energy consumption records assist the residents to make proactive energy utilization decisions. In addition, the MS is liable for real-time event generation. The smart home event generation is determined by in-house controls and remote user controls. In-house controls are generated by the MS to switch off active appliances in the absence of the user. However, the MS notifies the user and opts to wait for a time $\alpha_{T}$ to receive user approval. Hence, it helps the user to adjust different tasks of the smart home, i.e., air conditioner and refrigerator cooling and surveillance systems by approving or rejecting the MS-generated events. Once $\alpha_{T}$ expires, the MS generates decisions aligned with the HMI module. HMI incorporates previous smart home user experiences to make judgments corresponding to the future adjustments. Thus, the integration between HMI and MS guarantees that the unavailability of the user does not adversely affect the smart home operations. On the other hand, remote user controls are received by the MS via the smart gateway. The RESTful API translation facilitates the remote users to access the services through the Web. In general, remote controls actions include turning on appliances, turning off appliances, and monitoring real-time energy consumption. In addition, appliance-specific services are exposed to remote users via RESTful APIs, i.e., air conditioner cooling and water heating levels. The remote user request is made through the Web and received by the smart gateway, which sequentially, forwards it to the MS. Since the user initiates the event, the MS executes the request immediately and $\alpha_{T}$ is not involved.

**Figure 2:**
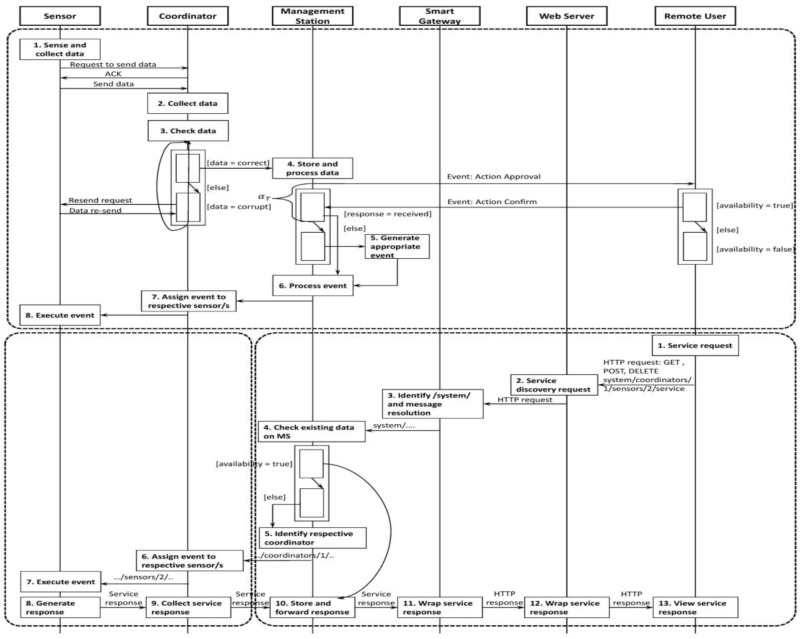
Event management system for in-house controls and remote smart home user controls.

At the arrival of the HTTP request, the MS selects the appropriate communication process. If the real-time information is available on the MS, it will directly process the HTTP request and generate the corresponding response. For example, if a user requests to get the status of an appliance, it is always available on the MS. Therefore, the MS responds to the user request immediately after processing. Nevertheless, in the absence of real-time data on the MS, it determines the corresponding ZigBee coordinator and ZigBee sensor to route the HTTP request. The identification is made easy with the self-describing HTTP request. At the completion of the service request, the MS sends the service response to the smart gateway wrapped as an HTTP response as shown in Figure 2. For example, assume that a remote user asks for the room temperature of a particular room in the smart home. Since the MS does not hold the real-time room temperature, it forwards the HTTP request to the respective coordinator and sensor to process the event accordingly.

The sensor performs service resolution and executes the event, i.e., getting the room temperature, and setting refrigerator cooling. Accordingly, the sensor generates the service response at the completion of the event. Consequently, the event response is sent back to the MS and it will traverse the smart gateway, RESTful API architecture, and Web server to serve the user request. While routing the response, the MS updates the appliances’ or sensors’ status and energy consumption parameters. Finally, the smart gateway wraps the service response that can be viewed by the respective user via a Web browser.

### 4.3. Service Representation over the Web

The Web provides ubiquitous application layer functionality to the smart home objects. Therefore, it is essential to provide a communication medium which directly links a home user to the smart home objects. In this section, we provide a Web-oriented architecture for smart home objects enabling them to speak the same language as other resources do on the Web. We have also thoroughly described the working mechanism of the restful API and show how the RESTful API can be used to integrate the smart home services with the Web. The dramatic rise of the connected devices coined the concepts of IoT and WoT. In general, the devices in the WoT adhere to two basic conditions i.e.: (1) implementing TCP/IP protocol stack over Ethernet or Wi-Fi networks and (2) implementing a Web server supporting the HTTP 1.1 protocol [3]. However, not all the embedded devices comply with the aforementioned two requirements due to computational, memory, and bandwidth limitations, despite the fact that the feasibility of adopting TCP/IP protocol in resource-constrained devices is increased with the recent research conducted on embedded devices, i.e., 6LoWPAN architectures, sensors, and devices with Wi-Fi support, embedded devices with built-in HTTP servers, and embedding Web servers on constrained devices. Nevertheless, the seamless integration of embedded devices into the Web is not guaranteed, since not all the embedded devices can meet the HTTP and TCP/IP demands. Thus, we propose a smart gateway architecture to compensate the HTTP and TCP/IP requirements of the non-adhering embedded devices, i.e., ZigBee coordinators, ZigBee sensors, and Bluetooth sensors. The smart gateway acts as a mediator between the Internet and smart things. Subsequently, the smart gateway provides transparent web processes, while hiding the internal details of the network from the remote smart home user. 

The proposed smart gateway consists of two layers, namely the Transport Module (TM) and the Device Service Module (DSM). The combination of TM and DSM creates the device controller for embedded devices operating on proprietary protocols such as ZigBee and Bluetooth. The bottom layer of the architecture enables the gateway to communicate via the appropriate communication protocol. In the proposed smart home scenario, coordinators and sensors use the ZigBee protocol for communication. ZigBee is a proprietary communication protocol that has been developed based on the IEEE 802.15.4 protocol. The DSM of the architecture resolves the proprietary service protocols of the devices and sensors. Further, device functionality mapping to RESTful APIs take place in the DSM layer. The smart gateway uses RESTful APIs to share ZigBee sensor data, which reports either environmental parameters, i.e., temperature, humidity, motion or data and functionalities of the attached embedded devices, i.e., device status or current energy consumption. Thus, the RESTful API architecture (RA) is introduced into the proposed architecture. The RA is responsible for binding the URI to the functionalities of virtual smart things on the Web, so that the remote smart home user can request home services, which generates a self-describing HTTP request via the MS of the house. The HTTP-enabled Web server is the other component of the proposed framework. The remote user request is received by the Web server and passed to the RA and smart gateway, respectively. Indeed, the smart gateway plays a vital role in the proposed WoT architecture along with RA and Web server, to ensure that non-web devices connect with the remote users via the Web. For example, the remote smart home user makes an HTTP request to get current temperature of the kitchen. The web server passes the HTTP request: /system/MS/coordinators/4/sensors/3/get_temp() received via the RESTful API. Sequentially, the appropriate sensor is located from the self-describing HTTP request. After identifying the corresponding sensor, it is passed to the DSM level, which translates the HTTP request into the ZigBee format. The TM captures the translated message, passes it to the MS of the smart home to process the service request as mentioned in the previous section. Similarly, the MS returns the service response and DSM converts the ZigBee response into a HTTP response with the aid of the RA in order to convey the response via the Web.

## 5. Performance Evaluation

The proposed framework is simulated for two different type of experiments. In one type of experiment, the entire proposed scheme is tested for the energy consumption of a household appliance. In another type of experiment, the communication of various home users with various household appliances is shown in the context of response time and transmission failure. 

### 5.1. Simulation Setup

A smart home architecture along with RESTful framework, MS, smart gateway, and a web server is simulated as shown in Figure 1. The MS and the smart gateway are installed on a Core I3 CPU 3.60 GHz computer. The RESTful framework and web server is placed on a separate computer equipped with the Windows Server 2008 operating system. The entire smart home scenario is tested in C# programming language. Each household appliance is attached with a ZigBee sensor. A sensor is a programming function which follows the IEEE 802.15.4 protocol architecture for communication. The coordinator node follows the structure of the Full-Function Device (FFD) that serve as the coordinator of a personal area network. The rest of the nodes are considered as Reduced-Function Devices (RFD) that are capable of modest resources and communication requirements. A star topology is followed by considering one FDD node in each room and kitchen attached to multiple sensors. The IEEE 802.15.4 does not define a network layer, therefore, we made the modification for routing the data following a multi-hop environment. Initially, each sensor node immediately connects with the coordinator and the coordinator is responsible of collecting and sending data to the MS. Similarly, the MS is enabled to follow a multi-hop routing strategy of IEEE 802.11n standard. An automatic topology adaptation is introduced to take care of the changes made in the network. For example, if a new node joins the network, the topology will change automatically to adapt to the arrival or depart of a node. Thus, it makes the system more dynamic and adaptive and it is able to handle a large number of nodes joining and leaving the network at once. Similarly, the sensing and controlling appliances functionality are added with different functions, i.e., collecting the data after a specific interval of time. In addition, we are not interested in the energy consumption of the sensors. Moreover, the RESTful framework operates as a separate program instead of installing it on each sensor. Therefore, the sensor nodes require energy only for sensing and transmitting information to the coordinator available at the shortest distance.

### 5.2. The Effect of the Proposed WoT-ESN Architecture

In order to test the proposed WoT-ESN architecture under a heavy load, a number of home users generate several requests to check the details of a household appliance through the Web. The frequency of the request generation is set to one request per minute for each home user. In the proposed smart home architecture, the number of users is less than the number of household appliances. Therefore, the response time in such situations is negligible for various requests. However, we tested a scenario where a greater number of users and a smaller number of appliances is considered. Moreover, the requests are passed to the respective sensors via MS following the multi-hop architecture of IEEE 802.11n network, even if the information is available in the MS. 

**Figure 3:**
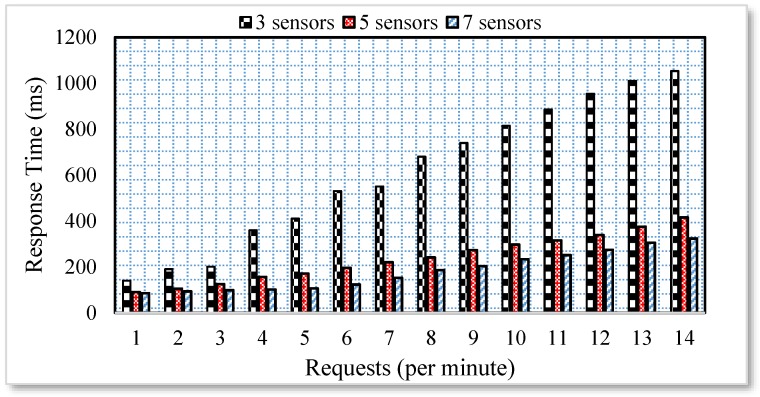
Response time analysis.

This experiment is performed for 10 min in various scenarios. In the first test, the webpage is placed on a separate computer while the MS and sensors are present in a different computer. In addition, both the webpage and sensor and MS are present on a different network to check the performance of the multi-hop concept in the proposed scheme. As shown in Figure 3, the users are sending requests to three, five, and seven household appliances. 

The results reveal that as the number of requests increases in the case of two household appliances, the response time also increases. However, with the increase of the number of household appliances, the response time decreases.

**Figure 4:**
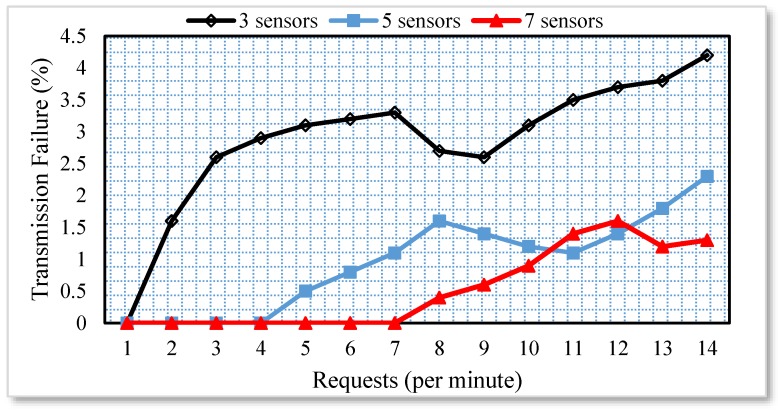
Transmission failure analysis.

The division of the requests in processes highly reduced the response time even if the requests are for the same household appliance. Moreover, each request is routed through different paths to check the effect of the multi-hop environment. Whenever the sensors are available at a closer distance to each other, there are greater chances of interference. Therefore, there is a high chance of failed attempts to send the requested data to the Web. In order to simulate a similar scenario, the sensor nodes available in the smart home scenario depicted in Figure 1 were divided into four groups. The groups 1, 2, 3 and 4 are available 3, 6, 9 and 12 m apart from each other. The group of sensors available 3 m apart from each other is highly affected by the interference and therefore, the failed transmission attempts in such scenarios are very high. The number of failed attempts when the number of requests reaches to 120/minute is more than 10%. However, the number of failed attempts significantly decreases when the physical distance between the sensors is more than 3 m. A similar scenario with three sensor nodes and multiple requests per minute is depicted in Figure 4.

**Figure 5:**
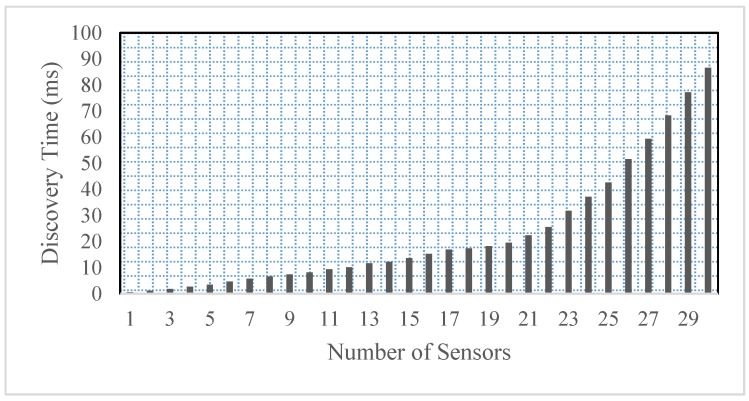
Device discovery analysis.

All the sensor nodes are operating in the same channel. It is clearly intuited from the results that the number of failed attempts are more than 3% of 120 requests/minute. However, these are the worst conditions, which is far better in real time scenarios by implanting the proposed scheme. The discovery time of all the sensors present in the smart home is computed as shown in Figure 5. Initially, the proposed system registers all the sensors node attached with each device in the Web server via the actuator and MS. However, if the number of sensors is more then it requires more time to load all the sensors into the framework. Therefore, it is very important to compute the discovery time of the devices present in the smart home.

An experiment is performed on discovering 30 sensors present in the proposed smart home scenario. The experiment reveals that if 20 sensors are turned on and discovered at once, it requires around 19 s to fully connect with the framework. However, the discovery time increases if more than 20 sensors are turned on at once.

### 5.3. Energy Consumption Analysis of the Household Appliances

**Figure 6:**
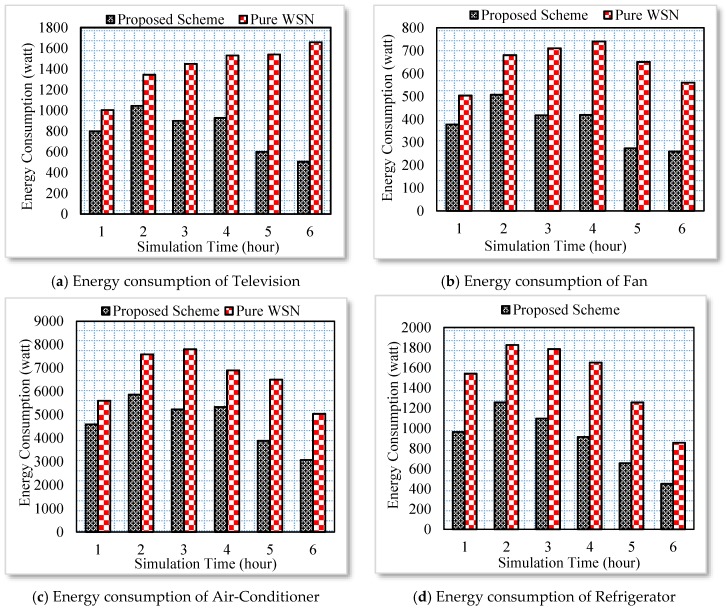
Energy consumption of various household appliances.

In this section, four different home users are interacting with various household appliances. They are programmed to randomly switch off and on a household device randomly. In order to be more realistic, the smart home user does not switch off most of the household appliances. Therefore, the user can check its status on the Web and can send a switch off request to the particular household appliance. A drop-down box with several records such as appliance status, energy consumption per day and month, and the number of times a device is switched off and on during a day is provided with each appliance. Those records help a user to tune the energy consumption of an appliance to optimal conditions. The proposed architecture is tested against a pure WSN. In the pure WSN scenario, the household appliances are directly controlled by the sensors. Whenever a user is not available in a scenario, the sensors automatically switch off devices. However, such systems do not perform actions based on the user context. Therefore, a pure WSN requires proper tuning of sensors for each new appliance. Such practices are only possible for controlling lights and home temperature. However, these WSN services cannot be integrated into real time scenario where the smart homes highly depend on user activities. In other words, the traditional WSN services cannot guarantee efficient services in the case ESN. The energy consumption of four different appliances, i.e., a television, fan, air-conditioner and refrigerator is shown in Figure 6a–d, respectively. In the case of proposed scheme the household appliances are controlled from the Web following a user context. However, in the case of pure WSN, the appliances are switched off and on considering the appliances’ operating time. The results show significant improvement in the context of energy consumption in the case of the proposed scheme. At average 15% to 20% energy is saved with the proposed system. 

**Figure 7:**
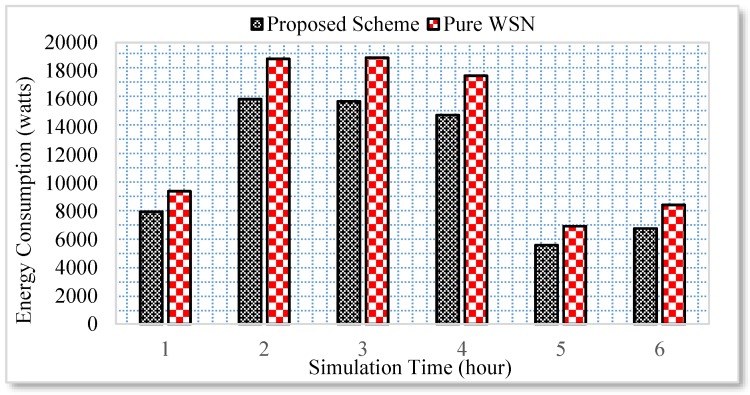
Energy consumption of entire home.

Similarly, the energy consumption of all the electronic appliances under the proposed scheme and a pure WSN scenario is compared for a duration of six hours. The user activity is random. For example, a user can go to any room or kitchen and switch on a household appliance. Unlike pure WSN, the household appliance is switched off following an approval from the user through the Web. However, if a user is not available at the moment, the user previous context is utilized in this case. The results reveal that an average of 15% energy is saved in the case of proposed scheme as shown in Figure 7. 

**Figure 8:**
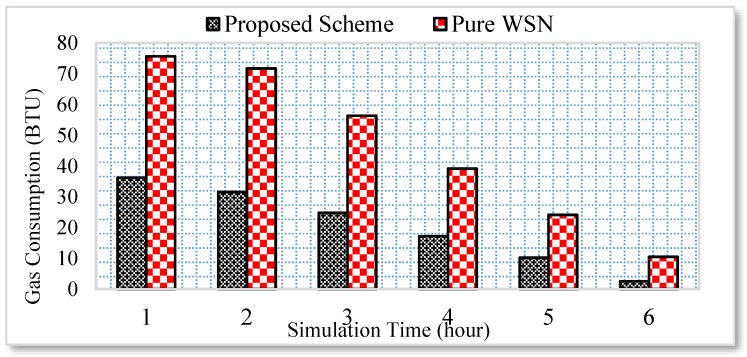
Gas consumption analysis.

**Figure 9:**
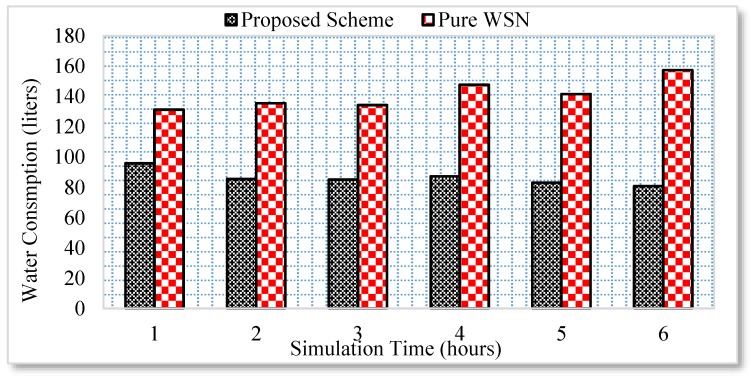
Water consumption analysis.

Similarly, the energy consumption of the gas burner and water consumption is significantly reduced, as shown in Figure 8 and Figure 9, respectively. 

A home user normally requires approximately 110 to 120 liters of water per day. Therefore, controlling the consumption of water optimizes the water usage per day. However, the traditional WSN architecture does not care about the user context and, therefore, a high amount of water is consumed due to the inappropriate usage. Similarly, the gas consumption is measured only for the burner in the kitchen. The rest of the gas consumption used for heating, etc. is not tested in this scenario. Initially, the home user used the kitchen for more time. However, with the passage of time the usage gets reduced. Thus, the energy consumption is significantly reduced following the proposed semi-automated model.

## 6. Conclusions

The integration of WoT with smart services is a challenging task, since it involves unification of heterogeneous technologies and services. However, a seamless and ubiquitous communication can be achieved by translating and mapping the data from these various technologies into a web representational format. HTTP is one of the conventional protocols mainly adopted for this type of services. However, HTTP has several issues in its use for the constrained application services. Therefore, in this paper, we have come up with a solution to link smart homes with the Web using REST architecture. Moreover, the data from heterogeneous devices need translation to the standard protocol in order to send it over IP. Thus, a smart gateway is used to map the data from the MS to the Web. Moreover, a web client can check and alter the status of a household appliance anytime by accessing the Web. The MS is designed in such a way that it can perform automatic actions if the smart home user is not available or does not have access to the Web. 

The entire smart integration with the Web architecture is simulated and tested for various functionalities. The energy consumption of the smart home appliances is evaluated in the case of a pure WSN and under the proposed scheme. The results show that adding user initiated semi-automated control of the appliances has optimized the energy consumption of household appliances. The proposed Web architecture is tested in the context of discovering the household appliances attached with the sensors, response time of the sensors, and transmission failure. The results reveal that the proposed scheme efficiently reduces the transmission failure by splitting the requests generated by the Web clients in multiple processes. Moreover, the response time is significantly enhanced by discovering the sensors in less time. Similarly, this smart home use case scenario can be further extended to various smart building scenarios, i.e., factories, warehouses, and smart grids to facilitate service accessibility via the Web.
